# Will psychology ever ‘join hands’ with disability studies? Opportunities and challenges in working towards structurally competent and disability-affirmative psychotherapy for energy limiting conditions

**DOI:** 10.1136/medhum-2023-012877

**Published:** 2024-06-24

**Authors:** Joanne Hunt

**Affiliations:** 1 Department of Women's and Children's Health, Uppsala University, Uppsala, Sweden

**Keywords:** disability, Health policy, psychotherapy, Ethics, Politics

## Abstract

Despite sustained efforts among critically informed scholars to integrate thinking from disability studies into psychology, the psy disciplines continue to largely neglect the lived experience of disabled people and overlook disability as a form of social inequity and valued culture. In this article, I make a renewed case for integrating thinking from disability studies into psy, in particular within the psychotherapy professions and in the case of ‘energy limiting conditions’, a grass-roots concept that includes clinically and socially marginalised chronic illness such as Long COVID. Drawing on my experience as a disabled practitioner, and situating this within extant literature on disability and psy, I take an autoethnographic approach to exploring opportunities and challenges in bridging the interdisciplinary divide. I argue that unacknowledged institutional ableism within psy reproduces and is reinforced by physical and attitudinal barriers for disabled practitioners and service users, engendering under-representation of disability in psychotherapy professions and lacunae in disability-affirmative conceptual resources. Additionally, I propose that hermeneutical lacunae are bolstered by documented defensive clinical practices pertaining to disability. After discussing a wealth of opportunities for integration offered by disability studies, and noting the institutional failure within psy to embrace disability-related demographic and epistemic diversity, I question whether ongoing epistemic and social exclusions within the psy disciplines constitute a case of ‘willful epistemic ableism’. Drawing on theorising vis-à-vis epistemic injustice and epistemologies of ignorance, I signal a form of systematic, actively maintained and structurally incentivised (motivated) non-knowing that results in collective failure among dominant groups to recognise established hermeneutical resources of the disabled community and allies. I conclude with suggestions of how this form of epistemic injustice might be mitigated.

## Introduction

 Broadly conceptualised, the psychotherapy professions encompass counselling, psychotherapy, clinical psychology, counselling psychology and clinical health psychology. Historically, these professions have had a difficult relationship with disability (Reeve 2006): burgeoning literature problematises psychotherapeutic theory, practice and policy for supporting individualistic, pathologising representations of disability, for largely ignoring structural and interpersonal disablism, and for failing to address barriers to training and services (eg, Goodley and Lawthom 2006; Olkin and Pledger 2003; Lund *et al* 2021; Ingham 2018). Despite such tensions, it is increasingly accepted within critical scholarship and activist work that disability studies can add significant value to psychotherapy practices and policy through transforming them in a socially just, disability-affirmative, sociopolitically and culturally cognisant direction (Goodley and Lawthom 2006; Goodley 2020; Olkin 2017; Reeve 2006, 2014; Prilleltensky 2009; Watermeyer 2013; Marks 1999).

Twenty years ago, US psychologists Rhoda Olkin and Constance Pledger published a paper advocating for greater integration of disability studies into (clinical) psychology (Olkin and Pledger 2003), posing the titular question: *Can disability studies and psychology join hands?* Their conclusion was that integration had not been achieved but was both possible and desirable, with the recommendation that:

To move closer, psychology must shed its emphasis on disability as abnormal, deviant, or special; join its sister disciplines in embracing disability studies; do everything within its power to foster the training and development of students with disabilities; and take the lead in ensuring that diversity includes disability and psychology includes diversity. (Olkin and Pledger 2003, 303)

As a disabled practitioner (within counselling and psychotherapy) and UK researcher working largely at the intersection of critical psychology and (critical) disability studies, I fully endorse these recommendations. Yet, 20 years later, despite parallel endeavours in the UK (eg, Goodley and Lawthom 2006; Reeve 2002, 2006, 2014; Simpson and Thomas 2015), psychology and psychotherapy have made remarkedly little progress on the road to joining hands with disability studies. In this article, I revisit interdisciplinary dialogues and tensions, and explore opportunities and challenges vis-à-vis integrating thinking from disability studies into psychotherapy. To increase the quality of my analysis, I draw on autoethnography (Ellis, Adams and Bochner 2011), locating my personal experience within the wider cultural context of psychology and disability. Adding to the originality of this contribution, and still drawing from personal experience, I explore opportunities and challenges within the arena of ‘energy limiting conditions’ or ‘energy limiting chronic illness’ (ELC) (Hale *et al* 2020, 2021; Evans *et al* 2023), a group of chronic illnesses that throw the multitude of tensions vis-à-vis psychology and disability into stark relief. Importantly, there is a notable dearth of literature on ELC and psychology through a disability-affirmative lens. Given my positionality as a UK practitioner-researcher, I focus primarily on the UK psychotherapy climate.

The structure of this article is as follows. First, I review academic literature capturing tensions and conversations between disability studies and psychotherapy. Noting that this literature largely overlooks diagnoses that can be conceptualised as ELC, I define this concept and discuss ELC in relation to psy and the wider sociocultural and political context. Second, I introduce autoethnography and position myself reflexively to the subject matter. Third, opportunities and barriers are discussed, interweaving autoethnographic elements with extant literature. In exploring opportunities for integration, I demonstrate that disability-affirmative conceptual resources exist that are well suited to psychotherapy work with ELC, yet these resources are largely unrecognised within psychotherapeutic circles. In making sense of this lack of recognition, and moving to consider barriers to integration, I problematise a climate of largely unacknowledged institutional ableism within psy that I suggest sustains a vicious cycle of social and epistemic exclusions. Given that this inequitable status quo is entwined with a neoliberal-ableist worldview that shows no signs of abating, I further conceptualise psy’s refusal to heed disabled knowledges: may we now contemplate a case of ‘willful epistemic ableism’ (a form of active, motivated ableist ignorance) within psy? Drawing from work on epistemic injustice and epistemologies of ignorance (Fricker 2007, 2016; Dotson 2012; Pohlhaus 2012, 2017; Medina 2013; Mills 2007), I signal a form of systematic, actively reproduced and structurally incentivised (motivated) non-knowing and related discrimination among individuals and institutions, resulting in collective failures among dominant groups to recognise the hermeneutical resources of disabled communities and allies. Assuming that willful epistemic ableism exists within psy, I then conclude with suggestions of how this refusal to engage may be addressed.

### Revisiting interdisciplinary encounters

The following narrative review seeks an interpretative understanding (Greenhalgh *et al* 2018) vis-à-vis evolving tensions and dialogues between disability studies and psychotherapy. Finding that ELC are largely missing from these dialogues, I then review literature that shines light on the troubled relationship between ELC and psychotherapy.

#### Historical culture clashes

Within disability studies, psychotherapy is positioned critically among the ‘psy’ disciplines: professional disciplines coalescing around psychology and psychiatry that promote normative and normalising discourse and practice in the psychological realm (Goodley 2017). Normativity inevitably produces exclusions, and psy has been criticised for conforming to oppressive abled (able-bodied and able-minded) ‘values’, norms and assumptions, while appealing to value-neutrality, apoliticality and evidence-based practice (Goodley and Lawthom 2006). More precisely, psy has been charged with promulgating disempowering, depoliticised, pathologising and individualistic constructions of disability consistent with the medical or tragedy (‘medico-tragic’) model, and thus colluding with the oppression of disabled people (Goodley and Lawthom 2006; Goodley 2020). Much foundational work within British disability studies and the UK disabled people’s movement, informed by the social model, has thus upended hegemonic psy representations of disability, instead conceptualising disability as a social construction and form of social oppression imposed on people with (physical) impairments (Oliver 1983; Oliver and Barnes 2012). Moreover, scholar-activists within disability studies have problematised knowledge hierarchies in psy, where practitioner-researchers are positioned as experts, and disabled service users or respondents are reduced to subjects of ‘expert’ practices (UPIAS 1976; Goodley and Lawthom 2006). In contrast, disability studies have adopted the disability rights ethos ‘nothing about us, without us’ and extend this ethos to encounters with psy (Goodley and Lawthom 2006; Olkin and Pledger 2003).

The above criticisms extend to the psychotherapy professions specifically. Here, a distinction might be made between therapeutically applied psychology (eg, clinical psychology) with its foundations in medicalised understandings of disability, and counselling and psychotherapy as a more heterogeneous and potentially more disability-affirmative group of approaches (see Hunt 2023a). However, my experience is that there is often very little distinction to be drawn in practice, with the exception of more ‘radical’ psychotherapeutic approaches (such as feminist therapy) that emerged as a reaction to the ‘three forces’ of psychoanalysis, cognitive-behaviourism and humanistic-existentialism (see Fleuridas and Krafcik 2019). While traditional psychoanalysis has been heavily criticised within disability studies (see Parsloe 2023), many of psychotherapy’s early articulations with disability were informed by a behaviourist (adapt-and-adjust) approach, an artefact of rehabilitation psychology informing dominant understandings of disability within psy (Olkin and Pledger 2003). From a social model perspective, rehabilitation psychology and kindred psychotherapies support the ‘medico-tragic’ model through their focus on adaptation to ableist environments or recovery (Oliver 1995; Oliver and Barnes 2012). Early attempts to integrate disability studies into psychotherapy, informed by the British social model, thus shifted attention away from impairment and focused near-exclusively on disabling social structures that were said to account for disabled people’s distress (Oliver 1995; Swain, Griffiths, and Heyman 2003).

These dynamics, in particular vis-à-vis the social model’s splitting of personal (impairment) and political (disability) and near-exclusive materialist focus on the latter, came under critique from multiple directions within disability studies (see Thomas 2007). Such critique would lead to an explosion of theoretical developments extending beyond the social model’s socioeconomic and political focus to encompass psychological, cultural, embodied and discursive analyses (Goodley 2013, 2017; Watermeyer 2013). Of particular relevance to this article, feminists contended that a more holistic and inclusive understanding of disability required theorising the lived, oftentimes distressing, realities of both disablism and impairment, not least since this would shine light on diverse (including gendered) perspectives somewhat sidelined by early social modelists and a male-dominated disabled people’s movement (Crow 1996; Morris 1991, 1996; Thomas 1999; Wendell 1996, 2001). Subsequent articulations between disability studies and psychotherapy generated more nuanced, embodied theorisations, including psychoemotional dimensions of disability (Reeve 2006, 2014; Simpson and Thomas 2015), which I discuss among opportunities for integration. Nevertheless, while such theorising continues to accumulate, there is little indication that psychotherapy is taking heed, notably within the UK.

An arguable exception to psychotherapy’s failure to engage with disability studies is the US ‘new paradigm’ (Olkin and Pledger 2003; Gill *et al* 2003; Prilleltensky 2009). This new paradigm moves away from dominant biological (medicalised, individualised) understandings of disability and points toward an interactional model, whereby disability is understood as a product of sociocultural influences in interaction with impairment. This has been accompanied by an increasing body of US literature promoting disability-affirmative psychotherapy practice and policy, and problematising barriers faced by disabled trainees (eg, Lund *et al* 2021; Brinkman *et al* 2023). However, despite the emergence of socioculturally and politically cognisant psychotherapies including feminist and transcultural modalities (Fleuridas and Krafcik 2019), disability continues to be under-represented as a minoritised group, culture and source of social inequity in the UK and beyond.

In light of the above, a principal point of divergence between disability studies and hegemonic psy (including dominant psychotherapies) can be articulated through the lens of structural competency, an awareness of how body/minds and healthcare systems are heavily shaped by structural forces such as institutions, legislation, policies, sociocultural and institutional norms, attitudes and infrastructure (Metzl and Hansen 2014). Disability studies could well be described as an inherently structurally competent discipline; however, hegemonic psy and psychotherapy—informed by a (neo)liberal individualism that overlooks sociostructural context—lack structural competency (Prilleltensky 2009; Hunt 2023a). An exception to this, and an acknowledged point of departure for productive dialogues between psychotherapy and disability studies, is the field of critical psychology (eg, Prilleltensky 2009; Goodley 2020; Goodley and Lawthom 2006). Of note, disability-affirmative therapy, developed by US psychologist Rhoda Olkin (Olkin 2007, 2017) is highly consistent with critical psychology and US new paradigm tenets, including its recognition of sociostructural context and unapologetically emancipatory ethos (see also Prilleltensky 2009). The term ‘disability-affirmative’ is also applied outside of Olkin’s work to describe approaches underpinned with an active prizing of disabled people, a non-ableist focus on flourishing as opposed to deficit, and recognition of disability as a culture that enriches society; my use of the term reflects this broader definition (eg, Swain and French 2000; Brinkman *et al* 2023; Halacre 2020).

Literature probing encounters between disability studies and psychotherapy has largely overlooked diagnoses that fall under the ELC umbrella, in particular those positioned as ‘medically unexplained’. I will shortly turn to exploring why this may be. Before doing so, it should be acknowledged that the concept of disability is highly heterogeneous. Foundational work within the UK disabled people’s movement focused on people with ‘physical impairments’ (UPIAS 1976), which in practice foregrounded ‘objective’, relatively non-fluctuating forms of corporeal diversity such as loss of a limb or paralysis (see Thomas 1999). However, as early feminist disability studies scholars recognised (Morris 1991, 1996; Wendell 1996, 2001), and notwithstanding debates on identity politics and language use (Spandler *et al* 2015; Shakespeare 2014), disability houses a multitude of permutations of body/mind diversity interacting with disabling environments, including intellectual disability, neurodiversity, chronic illness and mental distress. Although space constraints prevent further discussion, lack of disability-affirmative psy practice has been problematised in all cases (Bogart 2023; Goodley and Lawthom 2006; Spandler *et al* 2015). Energy impairment could well be considered a form of physical impairment; however, there are considerable differences between the clinical and social positioning of people with physical impairment as conceptualised by the early disabled people’s movement, and the positioning of ELC. This assertion is unpacked in what follows.

#### Energy limiting conditions

Developed by UK disabled researchers in co-production with disabled respondents (Hale *et al* 2020, 2021; Evans *et al* 2023), the concept of ELC delineates diagnoses that share energy impairment as a central experience and a substrate for disablism, most evidently in the form of societal minimisation and disbelief. ELC include ‘rare’ conditions such as Ehlers Danlos syndromes alongside more commonly occurring diagnoses such as migraine and fibromyalgia. Some diagnoses that sit within the ELC cluster are heavily clinically and societally ‘contested’ (sometimes referred to as ‘medically unexplained symptoms’ or ‘persistent physical symptoms’), such as myalgic encephalomyelitis/chronic fatigue syndrome (ME/CFS), multiple chemical sensitivity and chronic Lyme (Hale *et al* 2020, 2021). Long COVID, currently a site of clinical contestation, also sits well within the ELC umbrella (Evans *et al*. 2023). Frequently positioned as unworthy of disability status, ELC are subject to widespread societal and clinical disbelief, not least since they often fluctuate and are difficult to readily identify, thus defying prevalent (fixed, identifiable) stereotypes of impairment (Hale *et al* 2020; Evans *et al* 2023). Disbelief, or as disabled feminist scholar and person with ME/CFS Susan Wendell (1996) termed it, ‘epistemic invalidation’, then promotes ‘social abandonment’ (Wendell 1996): discriminatory barriers within the benefits system, health and social care systems, education and workplace, including physically inaccessible services and provisions, absence of appropriate services or deprioritisation within existing services. Such barriers inflict physical, psychological, social, occupational and economic harms (Hale *et al* 2020, 2021; Evans *et al* 2023; see also Bê 2016; Blease *et al* 2017; Lian and Robson 2017; Evans *et al* 2023; Merone *et al* 2022; Hunt 2022a).

Importantly, some diagnoses within the ELC cluster have politically troubling histories that bear great relevance to psychotherapeutic framing. While higher rates of mental health comorbidity relative to the general population have been reported among people with ELC and other long-term conditions (Daru 2023; Naylor *et al* 2012), debates have raged over the role that mental health, and psychosocial factors, should play in explanatory models, in particular in the case of ‘medically unexplained symptoms’ (see Lian and Robson 2017; Blease *et al* 2017). A concurrent trend to position ELC—most notably those lacking established biomarkers—as perpetuated by maladaptive psychology to be ‘corrected’ through positive mindset and increased activity (typically via cognitive-behavioural interventions) has been argued to be underpinned by a neoliberal-ableist project of welfare state demolition, privatisation of health and social services, and associated ‘work cure’ ideology (Frayne 2019; Hunt 2022a, 2022b; Clifford 2020; Stewart 2016; Rutherford 2007). This project heavily implicates psy, having been traced to alliances between the state, disability insurance and rehabilitation industries, and psychiatry (Rutherford 2007; Hunt 2022c, 2023b, 2024). Relatedly, the controversial UK PACE Trial (White *et al* 2011), where claims of effectiveness for cognitive-behavioural interventions in ‘treating’ ME/CFS were subsequently found to be overinflated (Marks 2017; Wilshire *et al* 2018), can be understood both as a product of these psy-corporate-state alliances and a facet of work cure ideology (Hunt 2022a, 2024).

Although most analyses of psy-corporate-state alliances in the realm of ELC have foregrounded ME/CFS (eg, Rutherford 2007; Hunt 2022a, 2022b, 2024), similar arguments have been extended to medically contested diagnoses such as multiple chemical sensitivity (Walker 2003). Indeed, near-parallel alliances can be discerned in the positioning of many long-term health conditions, including people living with mental distress, where established biomarkers are also lacking (Frayne 2019). In UK social policy, medically contested ELC and prevalent mental health diagnoses have been further clustered as ‘common health problems’, a category that constitutes an ever-expanding group of alleged ‘undeserving’ disabled people (Hunt 2022a, 2024). By positioning this group of disabled people as ‘not really disabled’ (Soldatic 2020), social protections such as a healthcare and welfare are limited or denied, pushing disabled people into the labour market whether or not they are capable of work (Clifford 2020; Stewart 2016; Hunt 2023b, 2024). Such manifestations of what can reasonably be termed biopower and the government of disability (Tremain 2015) are facilitated by a neoliberal-ableist psy complex and overarching ‘wellness industry’ which invites or coerces individuals to work on themselves in pursuit of the idealised (productive, self-sufficient, self-regulating) citizen (Goodley 2014). Neoliberal-ableism, a ‘logic that pursues the (hyper)normal’ (Goodley 2014, 21), recognises that neoliberalism and ableism are co-constituted, and that the idealised neoliberal subject-citizen is an abled subject. In this climate, ‘common health problems’ and ELC become the archetypal Other, systematically denied or devalued. The harms of this othering cannot be overstated.

In the wider disabled population, the above biopolitical logic, combined with a climate of increasing austerity and welfare conditionality, has been associated with distress, destitution, premature death and suicide within the wider disabled population (Stewart 2016; Clifford 2020). In the case of ‘common health problems’ (remembering this encompasses many ELC) such harms are compounded through systematic misrepresentation of moral character. That is, ‘objective’ physical impairments, conforming to stereotypes of ‘healthy disabled and permanently and predictably impaired’ (Wendell 2001, 21), tend to be socially constructed through a medicotragic model, where pity is a common social response (Oliver and Barnes 2012). In contrast, ELC are subject to construction through a lens of disability denial, coalescing in the social imaginary as the ‘work-shy benefits scrounger’ trope, where disability is framed as a lifestyle choice, and where disbelief and negative moral judgements are frequent social responses (Hunt 2024; Evans *et al*. 2023). Relatedly, whereas people with more ‘objective’ physical impairments tend to have their quality of life underestimated (Prilleltensky 2009), the inverse is true of ELC (see Hunt 2024). Moreover, while rehabilitation psychology-oriented approaches may be helpful for people with physical impairments such as amputation or spinal cord injury, these approaches may harm people living with energy impairment. This is particularly the case where postexertional malaise is a concern, as per ME/CFS and Long COVID (Décary *et al* 2021). In the case of ME/CFS, cognitive-behavioural psychotherapeutic rehabilitative interventions have traditionally focused on ‘correcting’ alleged ‘dysfunctional’ illness beliefs vis-à-vis the condition’s biomedical status, which in practice has meant encouraging (sometimes coercing) people to change their mindset and increase their activity levels, with widely documented evidence of harms (see Marks 2017; MEA 2015; Wilshire *et al* 2018). These harms, in the case of ME/CFS and beyond, are compounded by medical neglect and gaslighting, raising the possibility of iatrogenic trauma (Merone *et al* 2022; Fennell *et al* 2021). Such harms may be understood as a product of gendered disability denial, since many conditions that fall under the ELC umbrella (including ME/CFS and Long COVID) disproportionately impact women (Wendell 1996, 2001; Evans *et al* 2023).

ELC (and also mental distress) serve as an exemplar of how ‘historically, disability and femininity have been coupled’ (Goodley 2017, 46), as women’s body/minds have been pathologised through intersecting power systems of patriarchy, neoliberal-capitalism and experimental science, including hegemonic psychology (see Goodley 2017). These intersecting power systems have constituted an idealised citizen-subject that is both culturally ‘masculine’ and abled, prizing traits of rationality, independence and self-control (Showalter 1987; Wendell 1996; Goodley 2017). In the case of ‘medically unexplained’ ELC, healthcare discourse has been traced to hysteria, a diagnosis believed to largely affect women, allegedly indicative of an excess of ‘feminine’ emotionality (O’Leary 2018). Much of the discourse captured in biomedical and psychotherapy settings pertaining to ELC, including that of allegedly self-indulgent, demanding and complaining attention seekers encouraged by unduly supportive family (see Hunt 2023c; Blease *et al* 2017), is strikingly redolent of the attitude of the male-dominated psy professions towards putative ‘hysterics’ in the 1800s and early 1900s (Showalter 1987). Relatedly, clinical narratives of primary or secondary ‘gains from illness’, the suggestion that illness may be perpetuated by psychosocial or financial ‘advantages’, are prevalent within the ELC arena (Hunt 2023c) and are traceable to psy’s historical constructions of ‘hysterical’ or ‘neurasthenic’ women (Showalter 1987; Richman *et al* 2000). Moreover, documented problematics of informed consent regarding psychological ‘treatment’ for medically contested ELC highlights the historical and ongoing clinical tendency to undermine women’s agency (O’Leary 2018). Such considerations underline the fact that disability is intersectional (shaped by its interaction with other axes of oppression and privilege), and point towards a need for intersectional approaches to disability-affirmative practice (Brinkman *et al* 2023).

### Methods

In preparation for exploring opportunities and barriers to integrating thinking from disability studies into psychotherapy, I outline my choice of methodology. Autoethnography draws on the lived experience of the researcher as part of a larger group (here, both disabled people and psychotherapy practitioners) and seeks to situate this experience within wider sociocultural and political understandings, creating a multilayered account of the phenomenon investigated (Ellis, Adams and Bochner 2011). Of relevance to disability studies’ critique of psy, autoethnography emerged from a growing recognition of the limitations of dominant (experimental, hypothetic-deductive) methodologies within social sciences. Equally consonant with my stance as disabled researcher, it is a methodology that ‘treats research as a political, socially-just and socially conscious act’ (Ellis, Adams and Bochner 2011, n.p.), thereby disrupting hegemonic psy’s pretensions to objectivity and value-neutrality. Moreover, autoethnography facilitates the representation of minoritised perspectives to ‘break through the dominant representations of professional practice, creating new knowledges’ (Denshire 2014, 838). Through integrating autoethnographic elements into this article, I seek to surface neoliberal-ableist logic and power relations within psychotherapy practice and policy.

Autoethnography involves the researcher writing on lived experiences that ‘stem from, or are made possible by, being part of a culture’ (Ellis, Adams and Bochner 2011 n.p.); therefore, situating myself reflexively vis-à-vis disability and psychotherapy is important. For me, disability can be best understood through a pluralistic lens: as a form of social oppression imposed on people whose body/minds are deemed ‘impaired’, as a source of cultural and epistemic diversity, a valuable yet misrepresented social identity, a political position and historical community (Goodley 2017). I also understand disability through a biopolitical lens as an object of widespread endeavours to manage the alleged ‘problem’ posed by body/minds diverging from neoliberal-ableist norms and thus threatening governmental practice (Tremain 2015; see also Hunt 2023b).

In situating myself vis-à-vis psychotherapy and disability, I am struck by contradictions and inconsistences as documented by other disabled practitioners, especially as regards psychotherapy’s ostensible commitment to equality, diversity and inclusion (eg, Ingham 2018; Goodley and Lawthom 2006; Reeve 2002). For example, UK practitioner-researcher Donna Reeve noted in 2002 that training courses accredited by the British Association of Counselling and Psychotherapy failed to address disability while recognising other sources of social inequity and diversity (Reeve 2002). In 2018, UK practitioner-researcher Esther Ingham highlighted the ostensible commitment of the British Association of Counselling and Psychotherapy and British Psychological Society towards what could be termed structurally competent practice, while detailing personal experience of (dis)ableism in training, and noting theoretical and demographic under-representation of disability (Ingham 2018). The Health and Care Professions Council (HCPC), the UK regulatory body of clinical, counselling and health psychologists, states that all practitioner psychologists must ‘recognise the impact of culture, equality and diversity in practice and practise in a non-discriminatory and inclusive manner’ (HCPC 2023, 9). Similarly, the British Psychological Society exhorts all psychological practitioners to ‘move beyond the level of the individual’ and ‘address wider structural issues in society which maintain excluding processes and power differentials’ (BPS 2017, 36). Indeed, psychotherapy professional and regulatory bodies within the UK and beyond emphasise the importance of understanding marginally situated epistemologies, the need to recognise sociopolitical context, and the importance of inclusive, non-discriminatory practice (APA 2022; HCPC 2023; BPS 2017; BACP 2018). This ostensible commitment to structural competency and inclusion typically evaporates in the arena of disability, and I find the persistent disjuncture between ‘talk and action’ profoundly alienating, and emotionally and physically exhausting.

#### Patient and public involvement

There is no formal process of patient and public involvement. This article is a product of my lifelong lived experience as a disabled person combined with my practice experience, working with disabled people in mental health and wider therapeutic settings. I have worked with people harmed by psychotherapeutic modalities informed by individualistic psy models of disability that do not resonate with their lived experience, and have sustained harms personally as a patient. Additionally, I have experienced marginalisation and exclusions within my profession owing to structural (dis)ableism, while encountering other practitioners experiencing similar exclusions.

## Opportunities for integration

Chief among the contended benefits of interdisciplinary integration is an enhanced ability to theorise ‘the psychological impact of living with an impairment in a disabling society’ (Goodley 2020, 362). In practice, I have used both the social-relational model of disability and related concept of psychoemotional disablism (Thomas 1999, 2007; Reeve 2006, 2014) to facilitate such theorising and in seeking to adapt psychotherapeutic interventions in a structurally competent, disability-affirmative direction. This section draws on my experience and extant literature in exploring opportunities for integration of disability studies into psychotherapy.

### Disability-affirmative resources

The social-relational model of disability (Thomas 1999, 2007) offers a materialist-feminist inspired expansion of the British social model that holds great potentiality for integration into psychotherapy, and thus for structurally competent and disability-affirmative psychotherapy practice. This model incorporates the social model’s understanding of disability (disablism or disability discrimination) as macro-level and meso-level sociostructural barriers (employment policies, healthcare access, the benefits system and so forth) imposed on people with impairments (Oliver and Barnes 2012). Additionally, and inspired by feminist thought, it recognises the potential for discriminatory and disabling interactions at a ‘micro’ or interpersonal level (Thomas 1999). The social-relational model’s recognition of interpersonal relations as vehicles of both empowerment and disempowerment, growth and harm, bears obvious relevance to psychotherapy and is immediately amenable to integration into psy frameworks. Further, the model’s concept of structural disablism (sociostructural barriers) can productively challenge psychotherapists to develop structural competency, which in practice may include advocacy-related work, for example, supporting service users to assert their civil and human rights (Olkin 2007).

The related concept of psychoemotional disablism (Thomas 1999, 2007; Reeve 2006, 2014) acknowledges ‘the socially engendered undermining’ (Thomas 1999, 3) of the psychoemotional health of disabled people. This form of disablism operates from macro to micro level via pathways including the impact of sociostructural barriers, invalidating or otherwise distressing interactions with others, and internalised ableism (Reeve 2006, 2014). In this regard, the concept incorporates space not only to work with the psychoemotional impact of disempowering interpersonal and intrapersonal (intrapsychic) dynamics, with which practitioners will be familiar, but also the psychoemotional impact of navigating hostile sociopolitical and physical landscapes, which represents a structurally competent extension to dominant psychotherapy modalities. Importantly, the concept of psychoemotional disablism allows what may appear to be individual distress (typically understood as mental illness) to be reframed where appropriate as sociopolitically and relationally mediated distress, as the downstream effect of disempowering, disabling and exclusionary encounters with people and social structures. This reframing reflects the testimonies of people with ELC, where invalidating interactions with and exclusions from social structures (within health and social care, the workplace, education and benefits system) have been demonstrated to leave people with ELC feeling unwelcome, out of place and less worthy than others (Hale *et al* 2020; Bê 2016, 2019; Evans *et al* 2023; Merone *et al* 2022). Internalised ableism among people with ELC is discernible in reported conflicts around requesting social accommodations and communicating disability-related needs and experiences to others, and through documented feelings of low self-worth, low self-efficacy and identity-related stress (Hale *et al* 2020, 2021; Hunt 2022a; Wendell 1996). Reframing distress through a structurally competent lens also counters victim blaming and other facets of disability denial, which as previously referenced are well documented in psy practices involving ELC (see Hunt 2023c).

Importantly for psychotherapy work, the social-relational model of disability and concept of psychoemotional disablism allow for recognition of the body, with space carved for the embodied experience of illness (eg, Bê 2016; Reeve 2006, 2014; Thomas 1999, 2007). Again, these are elements in which psychotherapists should be well versed. Efforts to bring the body into disability studies, exemplified through the related concepts of ‘impairment effects’ (Thomas 2007) and ‘externally imposed impairment effects’ (Bê 2016), are particularly amenable to integration into psychology. ‘Impairment effects’ delineate the unavoidable impact of impairment on embodiment (Thomas 2007); this is important to acknowledge when working psychotherapeutically with ELC since, as previously noted, biological realities are often overlooked or downplayed. While impairment effects may ostensibly point towards a foregrounding of individualistic loss, acceptance and adjustment models of chronic illness (see Olkin and Pledger 2003; Reeve 2002, 2006), work from within medical sociology and disability studies to develop a sociology of impairment demonstrate how impairment is bound up with ‘geographies of privilege’ (Sherry 2016, 733) and is biosocial in character (Thomas 2007; Crow 1996). Such thinking underscores the need to work with individual body/minds and the social inequities that shape them. Following this line of thought, the term ‘externally imposed impairment effects’ (Bê 2016) describes impairment effects such as pain and fatigue created or exacerbated by social phenomena, including invalidating and exclusionary interactions with people or social structures: experiences of people with ELC underscore the applicability of this concept (Bê 2016; Evans *et al* 2023; Hunt 2022a). The concept of externally imposed impairment effects is thus structurally competent and allows practitioners to understand bodily restrictions and symptoms of ELC in interaction with a disabling society, as opposed to automatically reaching for individualistic explanations such as somatisation of unaddressed psychological conflict or thwarted social needs (see Hunt 2023c).

#### Seeking interdisciplinary resonance

In facilitating interdisciplinary integration, it has been noted that concepts within disability studies require ‘some resonance as a psychological construct based on psychological theory’ (Simpson and Thomas 2015, 1302), not least with a view to operationalising constructs for research. Accordingly, it has been suggested that research on the psychology of emotions might offer a theoretical bridge (Simpson and Thomas 2015). Additionally, and broadly consonant with the suggestion that social identity theory offers an interdisciplinary bridge (Dirth and Branscombe 2018), I have found the concepts of stigma and minority stress (straddling social psychology and sociology) to offer bridging opportunities (Link and Phelan 2001; Meyer 2003). Stigma has been conceptualised as a multifaceted construct where ‘labelling, stereotyping, separation, status loss, and discrimination occur together in a power situation that allows them’ (Link and Phelan 2001, 377). This ‘power situation’ points towards a broader sociostructural context facilitative of stigma, allowing a structurally competent (disability-specific) comparison to be drawn with (dis)ableism. It follows that concepts such as structural stigma (Hatzenbuehler 2016) and internalised or self-stigma (see Meyer 2003) broadly resonate with structural disablism and internalised (dis)ablism, respectively.

In complement, minority stress refers to chronic and sociostructurally mediated stress to which members of stigmatised social groups are exposed (Meyer 2003). The minority stress model helps to explain how stigma and discrimination engender negative psychological sequelae through distal stressors (sociostructural phenomena such as discrimination) and proximal stressors (individual responses to discrimination such as minoritised identity concealment and internalisation), thus resonating with the aforementioned pathways of psychoemotional disablism (Thomas 1999; Reeve 2006). The minority stress model, originally developed for work with lesbian, gay and bisexual populations, has been applied widely for working with racially minoritised persons and has more recently been extended to disability (Lund 2021). Although the model does not address the physiological sequelae of minority stress, thinking on how stigma and discrimination may become embodied (biologically embedded) can help to fill this gap (see Hunt 2022a), which in turn bears resonance with Bê’s concept of externally imposed impairment effects (Bê 2016). Taken together, such concepts and models can help explicate the impact that being marginally situated can have on physical and psychoemotional health, offering opportunities to build bridges between psychotherapy and disability studies.

While recommendations for specific practice interventions are beyond the scope of this article, I offer one example using a cognitive-behavioural therapy (CBT) approach. For the sake of transparency, I strongly object to CBT as a proposed clinical ‘treatment’ for ELC, and am wary of its resonance with the medical model and subsequent co-option into neoliberal welfare-to-work programmes (see Frayne 2019). Nevertheless, having worked in medicalised settings where CBT is *de rigueur*, I have sought to find a way to adapt CBT work in a structurally competent, disability-affirmative direction for disabled people who choose it. I have done this by drawing on principles from minority-group-affirmative CBT, sometimes referred to as culturally competent or culturally sensitive CBT (Austin and Craig 2015; Pantalone, Iwamasa, and Martell 2019), and from feminist approaches to CBT (Hurst and Genest 1995; Brown 2018). Thus, while having developed this approach prior to encountering disability-affirmative therapy (Olkin 2007, 2017), my general stance as a therapist is highly consonant with Olkin’s work (Olkin 2007, 2017). Affirmative CBT is often informed by the minority stress model (remembering the conceptual resonance with structural and psychoemotional disablism) and therapeutic activities include supporting service users to identify discrimination and prejudice from macro level to micro level, to address internalised oppression, and to draw on community strengths through seeking identity-affirming connections with others (Austin and Craig 2015). I have sought to develop disability-affirmative cognitive-behavioural interventions by formulating automatic thoughts, assumptions and core beliefs as a partial product of structural (dis)ableism and psychoemotional disablism, rather than (or in complement with) formulating at the individual level. Identifying and working with cognitions can then proceed at a macro level, challenging the veracity and value of discriminatory social representations of disability, and also at a micro level, challenging the service user’s internalisation of structural and interpersonal disablism (see David 2009). Integrating feminist perspectives (Hurst and Genest 1995; Brown 2018) can further balance traditional CBT’s individualistic, androcentric emphasis on rational cognitions with recognition that emotional responses are not only valid but often tied to disempowering sociocultural realities, with attention paid to power differentials within and outside of the therapeutic relationship.

While further research is clearly necessary to determine the potential supportive value of what I term disability-affirmative CBT outside of clinical anecdote, this example hopefully serves to demonstrate that possibilities for structurally competent adaptations exist for those who actively seek them. In this regard, it is pertinent to consider that ‘the dearth of evidence-based affirmative therapies is due, in a large part, to a lack of representation of people with disabilities in the field’ (Bogart 2023, 300). The above exploration of opportunities for integration demonstrates that a wealth of bridge-building possibilities exist. Yet, there is very little evidence of resources within disability studies being adopted by, or inciting interest among, psychotherapy practitioners, trainers or policy makers. The following section explores why this may be.

## Barriers to integration

While the majority of literature critiquing psychotherapy from a disability-affirmative perspective focuses on disablism (disability discrimination), my experience suggests that a primary challenge to interdisciplinary integration and thus to disability-affirmative psychotherapy policy and practice pertains to ableism, the ‘hidden referent’ of disability (Goodley 2017). Ableism has been defined as ‘cultural imaginary and social order centred around the idealised able-bodied and -minded citizen who is self-sufficient, self-governing and autonomous’ (Goodley 2020, 366–367). It can thus be understood as a barely perceptible power structure that shapes the psychosocial imaginary from which disablism emerges. In this section, I explore how institutional ableism in psy may produce, and be reinforced by, the ongoing failure within psy to heed disability-affirmative conceptual resources.

### Disavowed institutional ableism

The social imaginary of the psychotherapy professions could well be argued to be saturated with ableism, from theoretical concepts such as the idealised ‘fully functioning’ (self-determining, self-contained) person (Rogers 1961) and exquisitely rational subject (Ellis 1962), to everyday professional parlance such as being ‘blind’ or ‘deaf’ to culturally sensitive matters (see Proctor 2023). It has been noted that many psychotherapeutic models were developed during an era that normalised the segregation of disabled people (Halacre and Jalil 2017); disability was thus not present in the mainstream social and clinical imagination and this may have contributed to prevailing pathologising psy models of disability developing unchecked. The psy professions are beginning (belatedly) to recognise toxic legacies of racism, transphobia, homophobia and sexism (see Murphy 2017). However, it has been suggested that disablism is underacknowledged (Ingham 2018; Olkin and Pledger 2003; Reeve 2002, 2014), while the legacy of ableism or neoliberal-ableism is virtually absent from psychotherapy literature (but see Lucas Casanova *et al* 2022).

Conspicuously, articulations between psy and neoliberalism have been subject to increasing critical attention (eg, LaMarre *et al* 2019; Sugarman 2015), but chiefly without recognition that neoliberalism is heavily entwined with (dis)ableism, to the extent that ‘neoliberal-ableism’ (Goodley 2014) has become an established concept within disability studies. The impact of neoliberal-ableism on health and social systems as it pertains to ELC has already been outlined; it is thus crucial that psy recognises and addresses this influence when working with people living with ELC. My experience suggests that this climate of largely unacknowledged institutional ableism (or neoliberal-ableism) within psy engenders resistance to the inclusion of disabled people and disability-affirmative thinking. Indeed, inclusion of disabled people (demographic diversity) and integration of disability-affirmative thinking (epistemic or viewpoint diversity) are very likely interlinked, as the following discussion demonstrates.

Under-representation of disabled people in psychology and the psychotherapy professions persists, despite repeated problematisation from within disability studies and among disability-affirmative practitioners (Goodley 2020; Levinson and Parritt 2006; Stannett 2006; Reeve 2002;Lund *et al* 2021, 2023). Recent data from the British Association of Counselling and Psychotherapy suggest that disabled people represent just 11% of its members (Jackson 2022). Yet, as society’s largest minority group, disabled people represent approximately 24% of the UK population (Kirk-Wade 2023). Relatedly, British Association of Counselling and Psychotherapy literature demonstrates that disabled people continue to face considerable physical and attitudinal barriers to entering the professions, not only in terms of practitioners accessing training programmes, but also service users accessing therapy (Jackson 2022, 2023). Data from the USA support this picture, additionally indicating discrimination during interviews for training programmes, and higher than expected attrition rates among disabled trainees, with the suggestion that barriers might be pushing disabled people out of the profession (Lund *et al* 2021). Despite ongoing exclusions, it is axiomatic that disabled people can offer unique insights into disability through their lived experience, and that such insights are invaluable in developing disability-affirmative knowledges, practice and policy within psy and beyond (see Goodley and Lawthom 2006; Wendell 1996; Hale *et al* 2020; Evans *et al* 2023). Indeed, many of the models and concepts highlighted throughout this article, and resources I have found helpful for practice, are the product of disabled and chronically ill scholars and practitioners (Thomas 1999, 2007; Reeve 2002, 2006, 2014; Bê 2016; Olkin 2017). Demographic diversity and epistemic diversity are thus inter-related: barriers to disabled people accessing psychotherapy as practitioners or service users impact detrimentally on recognition of disabled and disability-affirmative knowledges, while lack of recognition of disability-affirmative knowledges impacts detrimentally on accessibility and inclusivity, reinforcing under-representation. The result is a vicious cycle of epistemic and social exclusions.

This institutional failure within psy to embrace demographic and epistemic diversity pertaining to disability is particularly underdocumented, and may be particularly entrenched, in the case of ELC. As previously explained, people with ELC often struggle to obtain recognition as disabled people with rights to accommodations and adjustments, largely owing to the unfavourable positioning of ELC in the clinical and social imaginary and its incongruence with prevalent stereotypes of impairment and disability (Bê 2016, 2019; Hale *et al* 2020; see also Wendell 1996). Additionally, common measures to improve physical inclusion (such as wheelchair accessible environments) are oftentimes ineffective or insufficient among people with ELC who are largely or entirely confined to the home; as I have learnt from lived experience, this can create further barriers to inclusion (Hunt 2023d). Moreover, the clinical tendency to psychopathologise ELC may engender further forms of discrimination and exclusion. For example, I have faced attitudinal barriers in psy training and work environments owing to conflation of ELC with assumed ‘poorly managed’ mental health diagnoses, suggesting a combination of institutional ableism and sanism.

#### Structural incentives, individual defences

In digging deeper into psy’s repeated failure to heed disability-affirmative resources, it could be argued that these epistemic exclusions engender a form of epistemic injustice, an injustice inflicted on individuals and groups in their capacity as knowers that is rooted in power differentials, structurally prejudiced conceptual resources, and ‘negative identity prejudice’ (Fricker 2007; Carel and Kidd 2014). In particular, hermeneutical injustice might be indicated, a structural form of epistemic injustice occurring when socioculturally dominant hermeneutical (meaning-making) frameworks fail to capture the knowledge, experiences and testimonies of a marginalised group (Fricker 2007; Carel and Kidd 2014; Blease *et al* 2017). In this case, neoliberal-ableism could be understood as a dominant hermeneutical framework that fails to capture disability-affirmative knowledges and culture. However, while seminal conceptualisations of hermeneutical injustice (Fricker 2007) assume that neither dominant nor marginally situated groups have the necessary resources to make sense of marginalised experiences, this clearly does not hold true at the disability-psy intersection. As this article demonstrates, disabled people and allies have developed a wealth of hermeneutical resources to understand disability, yet these are rarely acknowledged by the psychotherapy professions. Relatedly, psy’s refusal to engage with disabled and disability-affirmative knowledges surely suggests a degree of agency (practitioners are free to engage with disability-affirmative resources), yet seminal work on epistemic injustice conceptualises hermeneutical injustice as a purely structural phenomenon (Fricker 2007). Therefore, I propose that the concept of contributory injustice (Dotson 2012), whereby dominant groups refuse to acknowledge established hermeneutical resources of marginally situated groups, is more appropriate (see also Scully 2018; Mladenov and Dimitrova 2023; Peña-Guzmán and Reynolds 2019; Miller Tate 2019). This refusal has been termed ‘willful hermeneutical ignorance’ (Pohlhaus 2012) to emphasise that ‘such a refusal is *not* an inherent inability, but rather a willful act’ (Pohlhaus 2012, 729; see also Dotson 2012). Willful hermeneutical ignorance may thus constitute a significant barrier to integrating disability studies into psychotherapy, and also to addressing institutional ableism and its multitude of exclusions.

In relation to the above, it should be noted that ‘willful’ does not imply deliberate intention to do wrong (though such intent may exist). Willful hermeneutical ignorance can be understood as both a structural and individual phenomenon, whereby individual defences necessary to maintain ignorance (intersecting with epistemic vices) are reinforced by institutions and other social structures (Pohlhaus 2017; Dotson 2012; Medina 2013). According to this thinking, psy’s failure to engage could be considered structurally incentivised (motivated) by a neoliberal-ableist social order—a shared reality bias or form of ethnocentrism (see Anderson 2012)—that encourages complicity with injustice through encouraging dominant groups to ‘continue to misunderstand and misinterpret the world’ (Pohlhaus 2012, 716). Defensive behaviours among practitioners may then represent responses to the vague discomfort generated by what might be termed structural complicity (see Aragon and Jaggar 2018). Much work has been produced at the intersection of disability studies and critical social psychoanalysis with a view to ‘exposing non-disabled people’s unresolved, unconscious conflicts’ vis-à-vis disability (Goodley 2020, 362; see also Marks 1999; Watermeyer 2013; Goodley 2011, 2014, 2020; Wendell 1996), and more recently I have applied a similar lens to explore defensive clinical behaviours and disbelief in the ELC arena (Hunt 2023c). While the intricacies of these arguments vis-à-vis defensive practices are beyond the scope of this article, the implications are important: defensive (dismissive, disavowing, blaming, stereotyping) clinical behaviours may facilitate psychotherapists and other healthcare professionals in reinforcing structurally biased hermeneutical frameworks, even when other frameworks are available, and even when this may result in harm. Willful hermeneutical ignorance as it pertains to psy and disability, with or without intention to do wrong, can be deemed ethically culpable (Pohlhaus 2012; Medina 2013), not least since it can be considered to ‘represent a blameworthy failure to put the requisite effort or skill into knowing something one ought to know’ (Fricker 2016, 161).

## Conceptualising and addressing psy’s ‘refusal to engage’

The above discussions have revealed a wealth of conceptual and theoretical opportunities for integrating thinking from disability studies into psychotherapy, while also demonstrating the continued failure of psy to heed such hermeneutical resources. Given this persistent dynamic, and the harms wrought, I suggest that this persistent refusal to know requires further conceptualisation in disability-affirmative terms. The final section of this article thus opens with a question intended to provoke debate: might we now consider a climate of ‘willful epistemic ableism’ (a form of active, motivated ableist ignorance) within psychology and psychotherapy?

The term ‘epistemic ableism’ refers to the epistemic facets of ableism, and how ableism functions in part as an epistemology (Campbell 2009): how it reproduces dominant (abled) ways of knowing the world and how this impacts marginally situated, disabled knowers (see Peña-Guzmán and Reynolds 2019; Wieseler 2020). The notion of epistemic ableism thus sits well within epistemologies of ignorance, which ‘contend that ignorance, like knowledge, is produced and sustained through power relations and serves particular interests and values’ (Wieseler 2016, 86). The term ‘willful’ draws from (Pohlhaus 2012) and the concept of willful hermeneutical ignorance, while also recognising the dynamics of contributory injustice (Dotson 2012). My preference for willful epistemic ableism over ableist ignorance or non-disabled ignorance (see Wieseler 2016) reflects concerns regarding the concept of ignorance, notably its problematisation as a potentially ableist term on grounds of enlightenment categories of cognisance/ignorance mapping onto moral rights/wrongs (Tremain 2017; Simplican 2015). The concept of willful epistemic ableism seems to me to be more inclusive of different ways of ‘knowing’ or engaging in the world, and therefore more inclusive of the disabled community as a whole. Nevertheless, I draw from Medina’s concept of active ignorance (Medina 2013) and Mills’ concept of white ignorance (Mills 2007)—transposed into the disability space and with caveats regarding language—to further theorise this ongoing state of injustice perpetuated by psy.

Medina contends that active ignorance occurs when the non-knowing is underpinned with the active participation of subjects, facilitated via individual and collective defences and reinforced through structures that privilege some groups over others (Medina 2013). Akin to the concept of willful hermeneutical ignorance (Pohlhaus 2012), active ignorance is thus motivated (motivated by dominant groups’ investment in preserving the unequal power relations from which they benefit) and actively maintained. In helping to explain why some well-meaning practitioners might engage in willful epistemic ableism, Medina’s conceptualisation of active ignorance as ‘meta-ignorance’ is helpful (Medina 2013). Medina suggests that dominantly situated groups may be unaware of their ignorance (converging in the epistemic vices of closed-mindedness, laziness and arrogance) owing to deployment of defences, rendering such ignorance ‘capable of protecting itself’ (Medina 2013, 58). In complement, the work of [Bibr R79][Bibr R80] on white ignorance, a form of motivated ignorance rooted in white racial privilege, can further inform willful epistemic ableism, in particular given that ableism can be understood as a power system in broad parallel to (and in intersection with) whiteness or white supremacy (Bê 2019; Mills 2007). For example, following [Bibr R79][Bibr R80], it is likely that group interest plays a role in willful epistemic ableism, that non-disabled people generally have an interest in promulgating the ableist status quo (problematic non-disabled/disabled binary acknowledged). However, it also follows that some disabled people may reinforce such ableism, that some non-disabled people may actively work against it, that it can arise with and without discriminatory intent and with varying degrees of self-awareness (see Mills 2007; Fricker 2016; Wieseler 2016).

Thus, the concept of willful epistemic ableism may require further development to account for degrees of awareness and intent: ranging from injustices facilitated by a battery of defences and without deliberate intention to do wrong, to injustices that point toward *mens rea* (see Aragon and Jaggar 2018). In the former (likely far more common) case, willful epistemic ableism may be further elucidated through what has been termed ‘dysconscious ableism’ (Broderick and Lalvani 2017): ‘an impaired or distorted way of thinking about dis/ability […] that tacitly accepts dominant ableist norms and privileges’ (Broderick and Lalvani 2017, 895; see also King 1991). In the latter case, and given the earlier discussion of the role of psy-corporate-state alliances in furthering epistemic and social marginalisation of disabled people, it seems reasonable to consider the possibility (if only in rare cases) of collusion rather than complicity (see Aragon and Jaggar 2018). In all cases, it is reasonable to suggest that psy actors should have many of the requisite skills and levels of (self-)awareness to engage with and value the conceptual resources and knowledges of the people they purport to serve. The ‘psychological structures and social arrangements’ (Medina 2013, 57) that likely encourage willful epistemic ableism within psy have been discussed: the internalisation and reproduction of neoliberal-ableism which incentivises disability denial and devaluation, ensuing barriers to disabled people within the psy professions and barriers to disabled service users (with a resultant loss of disability-affirmative knowledge), and defensive practices which perpetuate the status quo.

Before moving to recommendations for mitigating willful epistemic ableism within psy, it is interesting to consider that such ableism may be intersected with other oppressive systems of power. For example, disability-affirmative hermeneutical resources as showcased in this article (eg, Thomas 1999, 2007; Reeve 2006, 2014; Bê 2016) arise broadly from within feminist disability studies and represent the epistemic labours of chronically ill and disabled women. Additionally, as already outlined, many (if not most) diagnoses that fall within the umbrella of energy limiting conditions are those that disproportionately affect women (Evans *et al* 2023). These dynamics, juxtaposed against the culturally masculine modes of knowledge historically produced by the psy disciplines (see Marecek and Lafrance 2021), indicate that psy’s continued overlooking of disability-affirmative frameworks may arise from androcentrism and/or sexism as well as ableism. Certainly, my experience as a disabled woman negotiating psy-related academic spaces is that some of these spaces are profoundly shaped by gendered and dis/abled knowledge hierarchies, and that these hierarchies contribute to the sidelining, or co-option and erasure, of disabled women’s epistemic labours. In this regard, it is important to note that (dis)ableism is shaped by further intersectional refusals to engage implicating race, ethnicity, class, gender and sexuality, giving rise to ‘polyphonic’ dynamics of power and resistance (Medina 2013, 2023).

Finally, and assuming that willful epistemic ableism exists, I proffer ways that we might address this form of epistemic injustice within psy. Since I have suggested that this type of ableism is both individually and structurally produced, it follows that both individual-level and structural changes are necessary (see Anderson 2012). Moreover, following Medina’s work on individual complicity with apparent structural injustices (Medina 2013), it is reasonable to suggest that there is a degree of individual as well as collective responsibility for mitigating willful epistemic ableism and its multitude of exclusions.

My first recommendation is that of greater emphasis on practitioner reflexivity in training, supervision and continued professional development, where reflexivity includes recognising the social situatedness and limitations of our ability to know and being open to other ways of knowing (see Hunt 2022a, 2023c). This is broadly consonant with Fricker’s contention that hermeneutical injustice can be ameliorated by a ‘reflexive critical sensitivity’ vis-à-vis the hermeneutical frameworks at play within encounters between dominantly situated and marginally situated knowledge producers (Fricker 2007, 7). It is also consistent with Medina’s suggestion that fighting epistemic injustices requires ‘meliorating epistemic habits’ (Medina 2013, 19). Defences among non-disabled practitioners against engaging with disability have already been referenced; additionally, disability-affirmative psychotherapists have noted that ethical practice necessitates critical self-reflection, in particular regarding (un)conscious conflicts around disability and impairment (Olkin 2007; Halacre 2020; Ingham 2018; Reeve 2002). Reflexivity is ostensibly highly prized in psychotherapy circles (eg, HCPC 2023; APA 2022), yet there appears to be a collective lack of reflexivity pertaining to disability, including ELC (Hunt 2023c). Given the importance of reflexivity vis-à-vis disability, I suggest it requires structural-level support, for example, by being formally integrated into training curricula and continued professional development. However, consistent with my suggestion that we might better speak of contributory injustice (Dotson 2012) rather than Fricker’s conceptualisation of hermeneutical injustice (Fricker 2007), I suggest that reflexivity is necessary but insufficient.

Following the above, I propose that the structural injustices involved in psy’s refusal to engage affirmatively with disability, together with the assumption of individual complicity in structural injustice (Medina 2013), point towards the need for a paradigm shift within psy. Much US disability-affirmative work, arising from or consistent with ‘new paradigm’ thinking (eg, Olkin and Pledger 2003) could set a precedent for similar initiatives within the UK. For example, US psychologists (Lund *et al* 2023, 185) suggest that breaking down (dis)ableist barriers within the psychotherapy professions requires:

[…] research centering the voices of multiple marginalized and disabled trainees, creating and reinforcing an antiableist culture at training programs and work environments, amplifying the voices and expertise of trainees and psychologists with disabilities and intersecting identities, and supporting disabled trainees and psychologists who may experience activism fatigue.

Consonant with these suggestions, I propose integrating formalised teaching of disability politics (including but not limited to the histories of oppression tied to ELC) into training curricula (Hunt 2022a, 2023e). Additionally, disabled practitioners (including those with ELC) must be included in the governance of psychotherapy professional bodies, to ensure policy is guided in a disability-affirmative and structurally competent direction (see Gill *et al* 2003). Relatedly, and following the suggestion of disabled British psychologists almost 20 years ago (Levinson and Parritt 2006), UK professional psychology and psychotherapy bodies should consider mentorship schemes for disabled practitioners, following the example of the American Psychological Association (APA 2024). Such bodies should also ensure that disability (understood through a structurally competent lens) is represented among their various divisions, sections and member networks. Lastly, and again following calls from UK disabled and disability-affirmative psychologists (eg, Levinson and Parritt 2006; Reeve 2002) disability equality training should be included in all professional training programmes, with the condition that such training is co-produced with disabled people, including people with ELC. Such recommendation supports Dotson’s contention that remedying contributory injustice requires ‘the ability to shift hermeneutical resources, which requires fluency in differing hermeneutical resources’ (Dotson 2012, 34). Disabled people, like all minoritised groups, are typically fluent in both dominant and subjugated hermeneutical resources and can thus act as translators whilst producing beneficial epistemic friction (see Medina 2013).

Finally, and valuing the work of activist-scholar-practitioners, I gravitate towards Medina’s suggestion that epistemic justice can only be achieved through a ‘sustained social activism’ (Medina 2013, 23). Such activism may involve actively resisting contributory injustice via strategic counter-refusals to engage with structures and subjects perpetuating this injustice, while ‘actively promoting the hermeneutical agency’ (Medina 2023, 233) of disabled psychologists, service users and allies. In this regard, greater solidarity between people with ELC and other minoritised groups harmed by psy may bear fruit. In particular, I recommend greater cultivation of solidarity and exploration of collective resistance practices between people living with mental distress (primary mental health diagnoses) and people with ELC (see Spandler *et al* 2015; Spandler and Allen 2018). Indeed, critical mental health literature, broadly situated at the intersection of disability studies, mad studies and critical psychology, offers a space that welcomes critical scrutiny of neoliberal-ableism and its constitution and governance of people with body/minds deemed ‘deviant’ and undeserving (eg, Frayne 2019; Spandler *et al* 2015). In this regard, and as highlighted elsewhere (Spandler and Allen 2018), discussions regarding restorative justice for people living with mental distress might be extended to the ELC community. Furthermore, and remembering that disability is intersectional, disabled activist-scholar-practitioners would likely benefit from forging alliances with those whose work foregrounds the defiant resistance of other ‘deviant identities’ (Harvey and Kotze 2022, 173; see also Turner 2024). Collectively, these recommendations are necessary to achieve ‘epistemic democracy’, defined as the ‘universal participation on terms of equality of all inquirers’ (Anderson 2012, 172), and recognised as the virtue of epistemic justice for institutions (Anderson 2012).

### Conclusion

In summary, this article has made a renewed case for integrating thinking from disability studies into psychology and psychotherapy, and particularly within the ELC arena. Taking an autoethnographic approach, tensions between the two disciplines have been revisited and opportunities to bridge the disciplinary divide have been discussed. Challenges, in the form of barriers to integration, have been conceptualised as an institutional failure to embrace disability-related demographic and epistemic diversity. Disavowed institutional ableism has then been contended to produce and be reinforced by this lack of diversity, giving rise to persistent marginalisation as experienced by disabled practitioners and service users, and perpetuating conceptually impoverished, disability-denying hermeneutical frameworks. Ongoing lacunae in practitioners’ hermeneutical resources have been theorised as both structurally incentivised (ultimately via neoliberal-ableism) and bolstered via defensive practices. Psy’s persistent refusal to engage with disabled and disability-affirmative knowledges has been theorised as willful epistemic ableism, and recommendations for addressing this form of epistemic injustice have been proffered.

If psychology and psychotherapy are genuine in the institutional and disciplinary commitment to ‘move beyond the level of the individual’ and ‘address wider structural issues in society which maintain excluding processes and power differentials’ (BPS 2017, 36), then disability must be included. Indeed, given the emergence of Long COVID and likelihood of a tidal wave of postpandemic disability (Hunt 2023e; Evans *et al* 2023), psychology must evolve in a disability-inclusive direction as a matter of urgency. Disability studies houses a theoretical and conceptual panoply that can greatly facilitate this endeavour. Whether disability will be emancipated from its position as the poor relation of equality, diversity, and inclusion practice and policy within psy remains to be established.

## Data Availability

No data are available.
